# Epilepsy-associated *CHD2* missense variants and optimization strategies for genetic diagnosis: a comparative analysis of algorithms

**DOI:** 10.3389/fneur.2025.1729387

**Published:** 2025-11-26

**Authors:** Yu-Jie Gu, Peng-Yu Wang, Qing-Qing Fu, Jia-He Lai, Xin Chen, Xiang-Hong Liu, Bao-Zhu Guan

**Affiliations:** 1 Department of Neurology, Ganzhou People's Hospital, Ganzhou, China; 2 Department of Neurology, Institute of Neuroscience, Key Laboratory of Neurogenetics and Channelopathies of Guangdong Province and the Ministry of Education of China, The Second Affiliated Hospital, Guangzhou Medical University, Guangzhou, China

**Keywords:** missense variant, *in silico* tools, *CHD2*, MutPred2, AlphaMissense, developmental and epileptic encephalopathy, optimizing genetic diagnosis

## Abstract

**Background:**

The *CHD2* gene is one of the most common causative genes of developmental and epileptic encephalopathy (DEE). With the advent of high-throughput sequencing, identifying *CHD2* variants has increased, necessitating evaluation of the gene-specific performance of widely used tools, as genome-wide benchmarks may mask such heterogeneity.

**Methods:**

The dataset of pathogenic and control *CHD2* missense variants was curated from ClinVar, HGMD, and PubMed databases. Tools included SIFT, SIFT4G, Polyphen2_HDIV, Polyphen2_HVAR, MutationAssessor, PROVEAN, MetaSVM, MetaLR, MetaRNN, M-CAP, MutPred2, PrimateAI, DEOGEN2, BayesDel_addAF, BayesDel_noAF, ClinPred, LIST-S2, ESM1b, AlphaMissense, and fathmm-XF_coding. The *in silico* tools were evaluated based on accuracy, sensitivity, specificity, positive predictive value (PPV), negative predictive value (NPV), Matthews correlation coefficient (MCC), F-score, and area under the ROC curve (AUC).

**Result:**

A total of 27 missense variants which were classified as pathogenic or likely pathogenic were used as a positive set, and 57 missense variants were used as a negative set. The top tools in accuracy are MutPred2, ESM1b, AlphaMissense, and PROVEAN. In terms of the MCC and F score, the higher degree was observed in MutPred2 and AlphaMissense (MCC score >0.8). ClinPred, AlphaMissense, and BayesDel_addAF had a higher AUC score (AUC > 0.99). SIFT, SIFT4G, Polyphen2_HDIV, Polyphen2_HVAR, ClinPred, and AlphaMissense scores exhibited a distinct bimodal distribution. While scores from other predictors showed a wider distribution range.

**Conclusion:**

Our study highlights the significant variation in the performance of different *in silico* tools for predicting *CHD2* missense variant pathogenicity. Given its overall performance, MutPred2 and AlphaMissense may be the preferred choice for clinical application in *CHD2*-associated DEE, providing possible reference in optimizing genetic diagnosis and classification of *CHD2* missense variants.

## 1 Introduction

Developmental and epileptic encephalopathy (DEE) represents a heterogeneous group of early-onset epilepsy disorders characterized by refractory seizures, accompanied by cognitive decline or regression associated with ongoing seizure activity (1). This debilitating condition significantly impacts the quality of life of patients and their families, necessitating robust diagnostic and therapeutic strategies. In recent years, significant advancements have been made in understanding the genetic underpinnings of DEE, with the identification of various causative genes, including *CHD2* (2).

The *CHD2* gene, located on chromosome 15q26, encodes the chromodomain helicase DNA binding protein 2, which is abundantly expressed in the brain, particularly in the neocortex, hippocampus, cerebellum, and olfactory bulb (3, 4). As chromatin remodelers, CHD2 proteins play crucial roles in gene regulation and neuronal development (2). Mutations in the *CHD2* gene have been implicated in a range of neurodevelopmental disorders, including DEE, autism spectrum disorder (ASD), intellectual disability (ID), and attention deficit hyperactivity disorder (ADHD) (2, 4–10).

With the advent of high-throughput sequencing technologies, variant screening in patients with DEE has led to the discovery of numerous *CHD2* variants, many of which are missense variants (2, 11–26). However, determining the pathogenicity of these variants remains a significant challenge due to the time-consuming and costly nature of functional evaluations. In this context, bioinformatics tools have emerged as indispensable resources for predicting the functional significance of variants (27, 28). These tools leverage various methodologies, such as sequence conservation, amino acid property analysis, and structural modeling, to classify variants into categories including “pathogenic/deleterious” or “benign/tolerant” (27, 29–41).

Despite the proliferation of *in silico* tools, their performance in predicting the pathogenicity of *CHD2* missense variants varies widely. Several studies have evaluated the accuracy, sensitivity, and specificity of these tools, with inconsistent results (42). Therefore, there is a pressing need for a comprehensive evaluation of the performance of these tools specifically for *CHD2* missense variants to guide clinical decision-making and improve patient outcomes.

In this study, we aimed to evaluate the prediction performance of 20 *in silico* tools for *CHD2* missense variants. By collecting a dataset of pathogenic and control variants, we assessed accuracy, sensitivity, specificity, positive predictive value (PPV), negative predictive value (NPV), Matthews correlation coefficient (MCC), *F*-score, and area under the receiver operating characteristic curve (AUC) of each tool. Our findings reveal significant differences in the performance of these tools in several aspects, with MutPred2 and AlphaMissense maybe the first line tool overall. This study provides valuable insights into the strengths and limitations of various *in silico* tools, facilitating more informed and accurate predictions of *CHD2* missense variant pathogenicity. Furthermore, our findings provide clues for clinical gene interpretation, highlighting the potential of *in silico* tools to improve the diagnosis and management of DEE and related neurodevelopmental disorders.

## 2 Materials and methods

### 2.1 Variants collection and analysis

To systematically evaluate the performance of *in silico* tools in predicting the pathogenicity of *CHD2* missense variants, variants were initially divided into two groups: pathogenic and control. Pathogenic variants were meticulously curated from the Human Gene Mutation Database (HGMD) and PubMed using the query: CHD2 AND (variant OR mutation OR polymorphism). Subsequently, a rigorous selection criterion was applied, included only those variants accompanied by clinical information, explainable origins for genetic diseases, and the American College of Medical Genetics and Genomics (ACMG) criteria. The control variants were retrieved from the ClinVar database, which were classified as “benign” or “likely benign” by ACMG criteria and reviewed by our panels. The data review cutoff date was August 31, 2025.

### 2.2 *In silico* prediction

A panel of 20 bioinformatics tools was selected for this study, chosen based on their widespread use and established effectiveness in predicting the functional impact of missense variants. The selection of these tools was based on three primary criteria: (1) inclusion in the ACMG variant interpretation guidelines or recommendation by clinical genetics consortia, (2) the availability of well-defined and recommended score thresholds for pathogenicity classification; (3) the ability to generate a valid score for at least 80% of the variants included in this study; (4) or demonstrated performance in published comparative studies of variant pathogenicity prediction. All prediction scores were obtained from the pre-computed databases (62) to ensure reproducibility. These tools included:

1) SIFT (Sorting Intolerant from Tolerant): a tool utilizes sequence conservation and amino acid properties to predict the impact of amino acid substitutions on protein function (31).
2) SIFT 4G: a faster version of SIFT (Sorting Intolerant from Tolerant), capable of providing predictions for a large number of organisms (41).
3) PolyPhen-2_HDIV: the HumDiv-trained version (HDIV) is optimized for assessing rare alleles potentially involved in complex traits, fine-mapping regions identified through genome-wide association studies, and evolutionary analyses where even mildly deleterious variants are considered damaging (43).
4) PolyPhen-2_HVAR: the HumVar-trained version (HVAR) is specifically designed for the diagnosis of Mendelian diseases, aiming to distinguish strongly deleterious mutations from the remaining human variation, including the presence of mildly damaging alleles (43).
5) Mutation Assessor: a tool predicts functional impact based on the evolutionary conservation of affected amino acids in protein homologs (34).
6) PROVEAN (Protein Variation Effect Analyzer): a tool combines evolutionary conservation, neural network models, and the BLOSUM62 scoring matrix to assess variant impact (28).
7) MetaSVM: an ensemble-based predictor for missense variant deleteriousness. It integrates nine individual deleterious prediction scores along with the maximum minor allele frequency (44).
8) MetaLR: an ensemble scoring method for deleterious missense mutations, which demonstrated the value of combining information from multiple orthologous approaches (44).
9) MetaRNN: a deep recurrent neural network–based ensemble models that integrate 28 high-level annotation features, including multiple functional prediction scores, evolutionary conservation metrics, and allele frequency information, to predict the pathogenic likelihood of human nonsynonymous SNVs and non-frameshift indels (45).
10) M-CAP: a pathogenicity classifier for rare missense variants that integrates existing prediction scores and additional genomic features within a high-sensitivity model, optimized for clinical use (46).
11) MutPred2: a machine learning–based predictor that employs an ensemble of neural networks trained on large sets of pathogenic and putatively neutral variants (47).
12) PrimateAI: a tool uses deep neural networks to predict the clinical impact of human mutations, incorporating primary sequence and protein structure information (40).
13) DEOGEN2: a tool integrates heterogeneous information about molecular effects, domains, gene relevance, and protein interactions to predict variant deleteriousness (33).
14) BayesDel_addAF/BayesDel_noAF: a Bayesian ensemble-based meta-predictor that estimates the deleteriousness of both coding and non-coding variants, including single-nucleotide variants and small insertions or deletions (48).
15) ClinPred: a machine learning–based predictor for disease-associated missense variants that integrates existing pathogenicity scores with population allele frequency from gnomAD database and is trained on ClinVar data to achieve highly accurate and robust pathogenicity classification across diverse disease contexts (49).
16) LIST-S2: the successor to LIST, quantifies conservation across species and predicts variant deleteriousness, not limited to human sequences (32).
17) ESM1b: a 650-million-parameter protein language model trained on 250 million protein sequences from diverse organisms using a masked language modeling objective, in which randomly masked residues are predicted based on their surrounding sequence context (50).
18) AlphaMissense: an adaptation of AlphaFold, which trained on databases of population frequencies of human and primate variants, incorporating structural context and evolutionary conservation as parameters for predicting missense variant pathogenicity (27).
19) FATHMM-XF: the enhanced version of functional analysis through hidden Markov models with additional features, improving predictions for single-nucleotide variants across the genome (35).

### 2.3 Evaluation of predictive performance

To accurately assess the predictive performance of these *in silico* tools, we calculated a series of critical metrics, including true positives (TP), true negatives (TN), false positives (FP), and false negatives (FN). From these metrics, we derived additional indices such as accuracy, sensitivity, specificity, positive predictive value (PPV), and negative predictive value (NPV). Furthermore, we computed the *F*-score, which balances precision and recall, and the Matthews correlation coefficient (MCC), which measures the quality of binary classifications. The *F* score was defined as $\frac{2\mathrm{PR}}{P+R}$ , where precision (P)= $\frac{\mathrm{TP}}{\mathrm{TP}+\mathrm{FP}}$ and recall (R)= $\frac{\mathrm{TP}}{\mathrm{TP}+\mathrm{FN}}$ .

The Matthews correlation coefficient (MCC) score was calculated using the following equation $\frac{\mathrm{TP}\times \mathrm{TN}-\mathrm{FP}\times \mathrm{FN}}{\sqrt{\left(\mathrm{TP}+\mathrm{FP})(\mathrm{TP}+\mathrm{FN})(\mathrm{TN}+\mathrm{FP})(\mathrm{TN}+\mathrm{FN}\right)}}$ , with the score ranging from −1 to 1; −1 indicates a completely wrong binary classifier, while 1 indicates a completely correct binary classifier.

Additionally, ROC curves were plotted for each tool, with the pathogenic missense variants serving as the gold standard for positive samples and the control missense variants with no relevant phenotypic reports serving as the gold standard for negative samples. The area under the ROC curve (AUC) provides a quantitative measure of the overall predictive performance of each tool.

### 2.4 Statistical analysis

Statistical analyses were conducted using R (4.5.1). For comparisons between two independent samples, the choice of statistical test was contingent upon the normality of the data. Normality was assessed using the Shapiro–Wilk test. If the data followed a normal distribution, Student’s t-test was employed. Conversely, for non-normally distributed data, the Mann–Whitney *U* test (also known as the Wilcoxon rank-sum test) was utilized. A *p*-value < 0.05 was considered statistically significant for all comparisons.

## 3 Results

### 3.1 Performance evaluation of *in silico* tools

In this study, we assessed the predictive capabilities of 20 different *in silico* tools specifically tailored for analyzing *CHD2* missense variants. Variants were meticulously curated from reliable sources: pathogenic variants were sourced from HGMD and PubMed, supported by robust clinical data, while control variants were retrieved from the ClinVar database (2, 11–26). This meticulous variant collection yielded a dataset comprising 27 pathogenic and 57 control variants, totaling 84 variants for comprehensive analysis (Supplementary Table S1).

**Figure 1:**
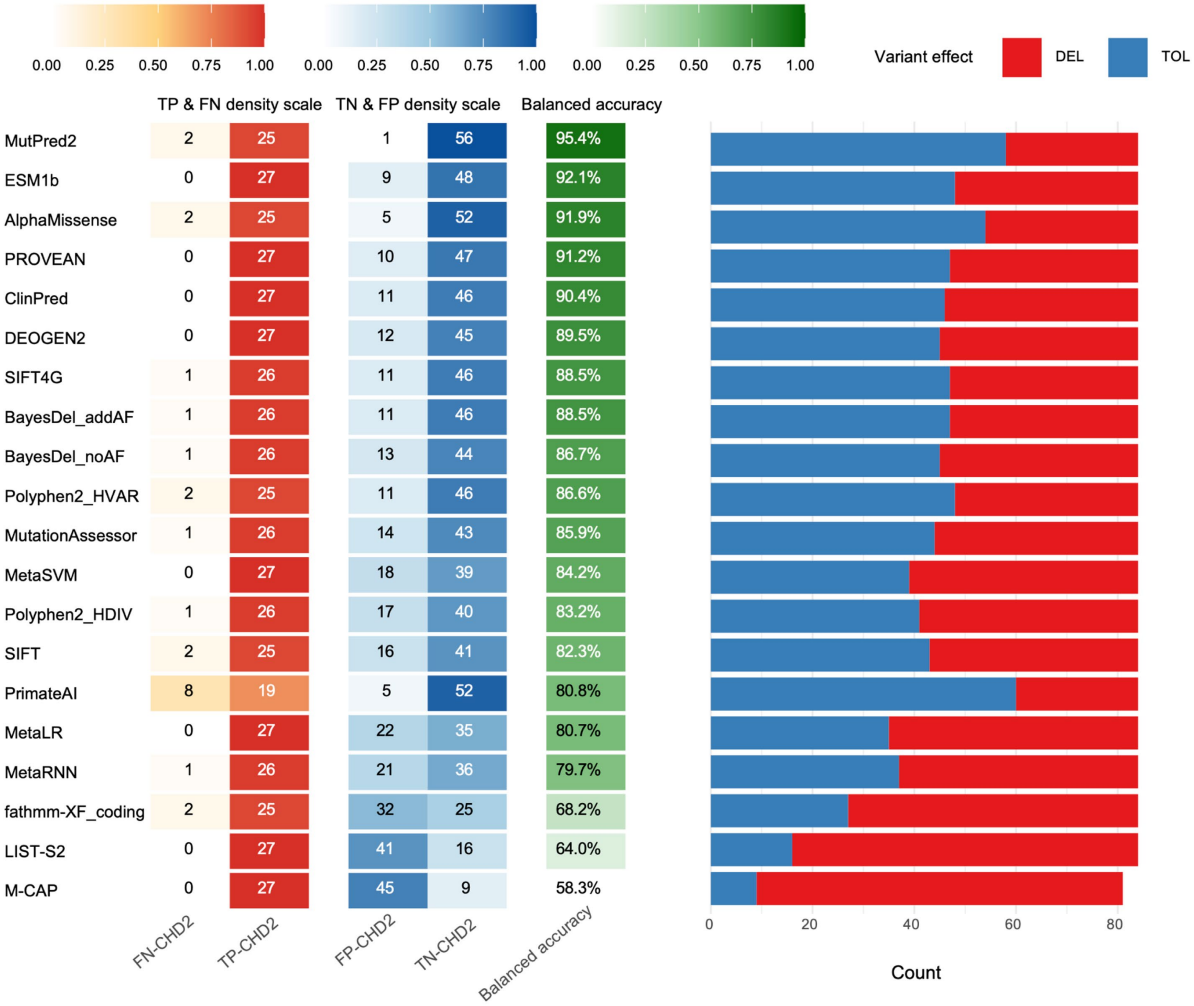
Performance evaluation of algorithms. For each tool, the left heat-map panel reports the rates of true positives (TP) and false negatives (FN) for pathogenic variants and true negatives (TN) and false positives (FP) for control variants. The middle column gives the resulting balanced accuracy (balanced acc.), which was used to rank the tools from top to bottom. The right stacked bar shows the proportion of variants each algorithm called deleterious (DEL, red) or tolerated (TOL, blue) using recommended thresholds.

Our analysis revealed substantial variability in the performance of these tools across various metrics (Figure 1 and Table 1). Accuracy, a crucial benchmark, ranged from a low of 58.3% (M-CAP) to a remarkable high of 95.4% (MutPred2). This striking difference underscores the importance of selecting the appropriate tool for variant interpretation. Sensitivity and specificity were evaluated, which are equally pivotal in assessing tool performance. Sensitivity, reflecting the ability to correctly identify pathogenic variants, varied from 70.4% (PrimateAI) to 100% (PROVEAN, MetaSVM, MetaLR, M-CAP, DEOGEN2, ClinPred, LIST-S2, and ESM1b). Conversely, specifically, indicating the capacity to accurately discern control variants, ranged from 16.7% (M-CAP) to 98.3% (MutPred2).

**Table 1:** Performance of *in silico* tools for the prediction of *CHD2* missense variants.

Software	TP	TN	FP	FN	Accuracy	Balanced accuracy	Sensitivity	Specificity	PPV	NPV	MCC	*F*-score
SIFT	25	41	16	2	0.7857	0.8226	0.9259	0.7193	0.6098	0.9535	0.6028	0.7353
SIFT4G	26	46	11	1	0.8571	0.885	0.963	0.807	0.7027	0.9787	0.7244	0.8125
Polyphen2_HDIV	26	40	17	1	0.7857	0.8324	0.963	0.7018	0.6047	0.9756	0.6211	0.7429
Polyphen2_HVAR	25	46	11	2	0.8452	0.8665	0.9259	0.807	0.6944	0.9583	0.6917	0.7937
MutationAssessor	26	43	14	1	0.8214	0.8587	0.963	0.7544	0.65	0.9773	0.6708	0.7761
PROVEAN	27	47	10	0	0.881	0.9123	1	0.8246	0.7297	1	0.7757	0.8438
MetaSVM	27	39	18	0	0.7857	0.8421	1	0.6842	0.6	1	0.6407	0.75
MetaLR	27	35	22	0	0.7381	0.807	1	0.614	0.551	1	0.5817	0.7105
MetaRNN	26	36	21	1	0.7381	0.7973	0.963	0.6316	0.5532	0.973	0.5593	0.7027
M-CAP	27	9	45	0	0.4444	0.5833	1	0.1667	0.375	1	0.25	0.5455
MutPred2	25	56	1	2	0.9643	0.9542	0.9259	0.9825	0.9615	0.9655	0.9177	0.9434
PrimateAI	19	52	5	8	0.8452	0.808	0.7037	0.9123	0.7917	0.8667	0.6368	0.7451
DEOGEN2	27	45	12	0	0.8571	0.8947	1	0.7895	0.6923	1	0.7393	0.8182
BayesDel_addAF	26	46	11	1	0.8571	0.885	0.963	0.807	0.7027	0.9787	0.7244	0.8125
BayesDel_noAF	26	44	13	1	0.8333	0.8674	0.963	0.7719	0.6667	0.9778	0.6882	0.7879
ClinPred	27	46	11	0	0.869	0.9035	1	0.807	0.7105	1	0.7572	0.8308
LIST-S2	27	16	41	0	0.5119	0.6404	1	0.2807	0.3971	1	0.3338	0.5684
ESM1b	27	48	9	0	0.8929	0.9211	1	0.8421	0.75	1	0.7947	0.8571
AlphaMissense	25	52	5	2	0.9167	0.9191	0.9259	0.9123	0.8333	0.963	0.817	0.8772
fathmm-XF_coding	25	25	32	2	0.5952	0.6823	0.9259	0.4386	0.4386	0.9259	0.3645	0.5952

FN, false negatives; FP, false positives; MCC, Matthews correlation coefficient; NPV, negative predictive value; PPV, positive predictive value; TN, true negatives; TP, true positives.

To delve deeper into the predictive prowess of these tools, we examined additional metrics such as PPV and NPV (Table 1). PPV, signifying the proportion of true positives among all predicted positives, ranged from 37.5% (M-CAP) to 96.2% (MutPred2). Similarly, NPV, representing the proportion of true negatives among all predicted negatives, spanned from 86.7% (PrimateAI) to 100% (ESM1b, PROVEAN, ClinPred, DEOGEN2, MetaSVM, MetaLR, LIST-S2, M-CAP, and SIFT4G).

To further gauge the quality of binary classifications produced by these tools, we calculated the Matthews correlation coefficient (MCC) and F-score. Both metrics provided consistent rankings, with MutPred2 topping the charts, exhibiting an MCC of 0.918 and an F-score of 0.943.

### 3.2 ROC curves for the *in silico* tools

**Figure 2:**
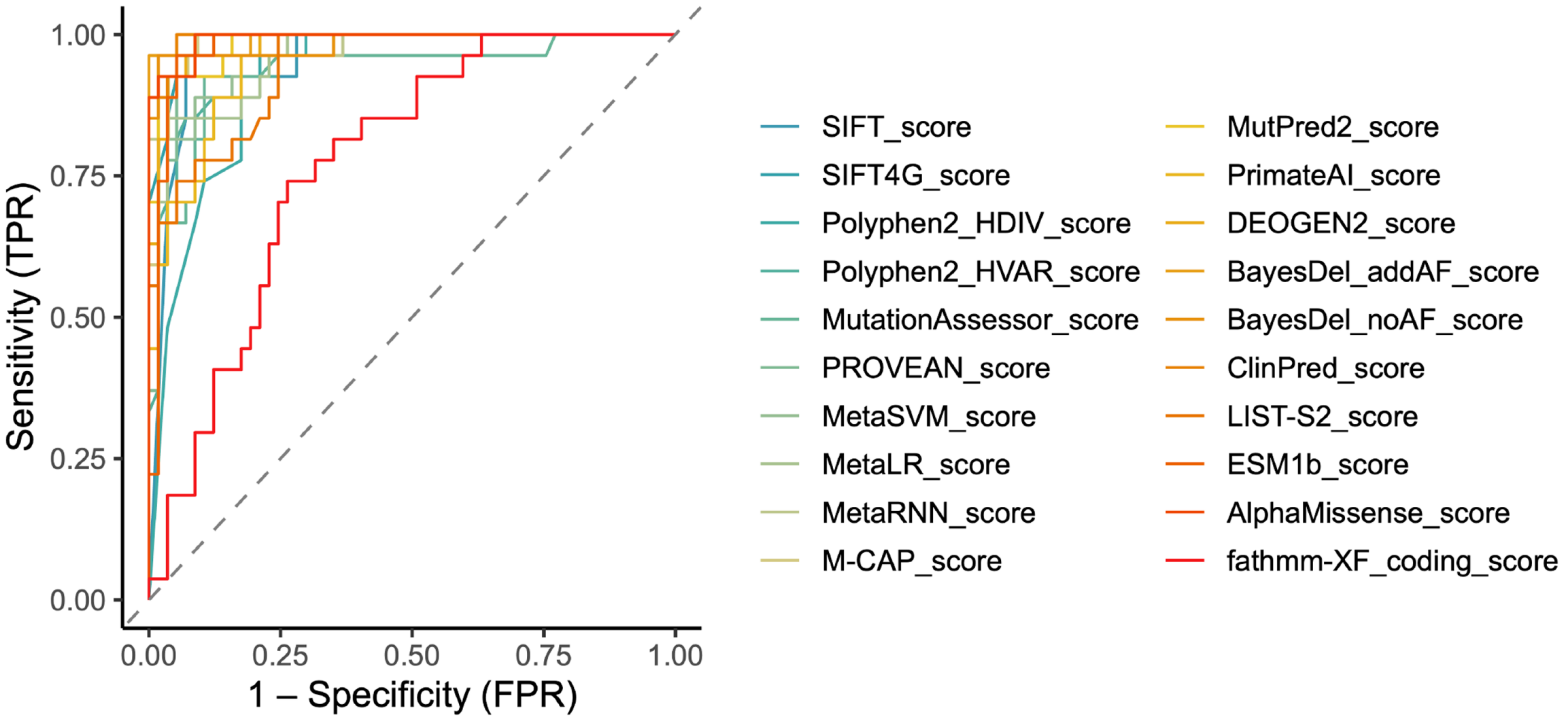
Receiver operating characteristic curve (ROC) performance and optimal thresholds of *in silico* tools for *CHD2* variants. Combined ROC curves showed each color is one tool, and the dashed line meant no discrimination. All curves are provided in Supplementary Figure S1.

To visually represent and compare the predictive performance of the tools, we generated ROC curves. The area under the curve (AUC), a quantitative measure derived from the ROC curve, provides an overall assessment of a tool’s diagnostic accuracy. Consistent with our previous findings, the ROC curve indicated that ClinPred (0.9948) emerged as the preeminent tool with an AUC value of 0.9948, followed by AlphaMissense (0.9929), BayesDel_addAF (0.9929), M-CAP (0.9856), MutPred2 (0.9851), BayesDel_noAF (0.9851), SIFT4G (0.9838), MetaRNN (0.9825), DEOGEN2 (0.9825), PROVEAN (0.9779), ESM1b (0.9773), MetaSVM (0.9734), MetaLR (0.9623), Polyphen2_HVAR (0.9561), SIFT (0.9539), PrimateAI (0.9526), LIST-S2 (0.9418), MutationAssessor (0.9379), Polyphen2_HDIV (0.9230) and fathmm-XF_coding (0.7745) (Figure 2). These AUC values reflect the exceptional ability of ClinPred, AlphaMissense, BayesDel_addAF to discriminate between pathogenic and control variants.

### 3.3 Distributions of the prediction scores for the *in silico* tools

**Figure 3:**
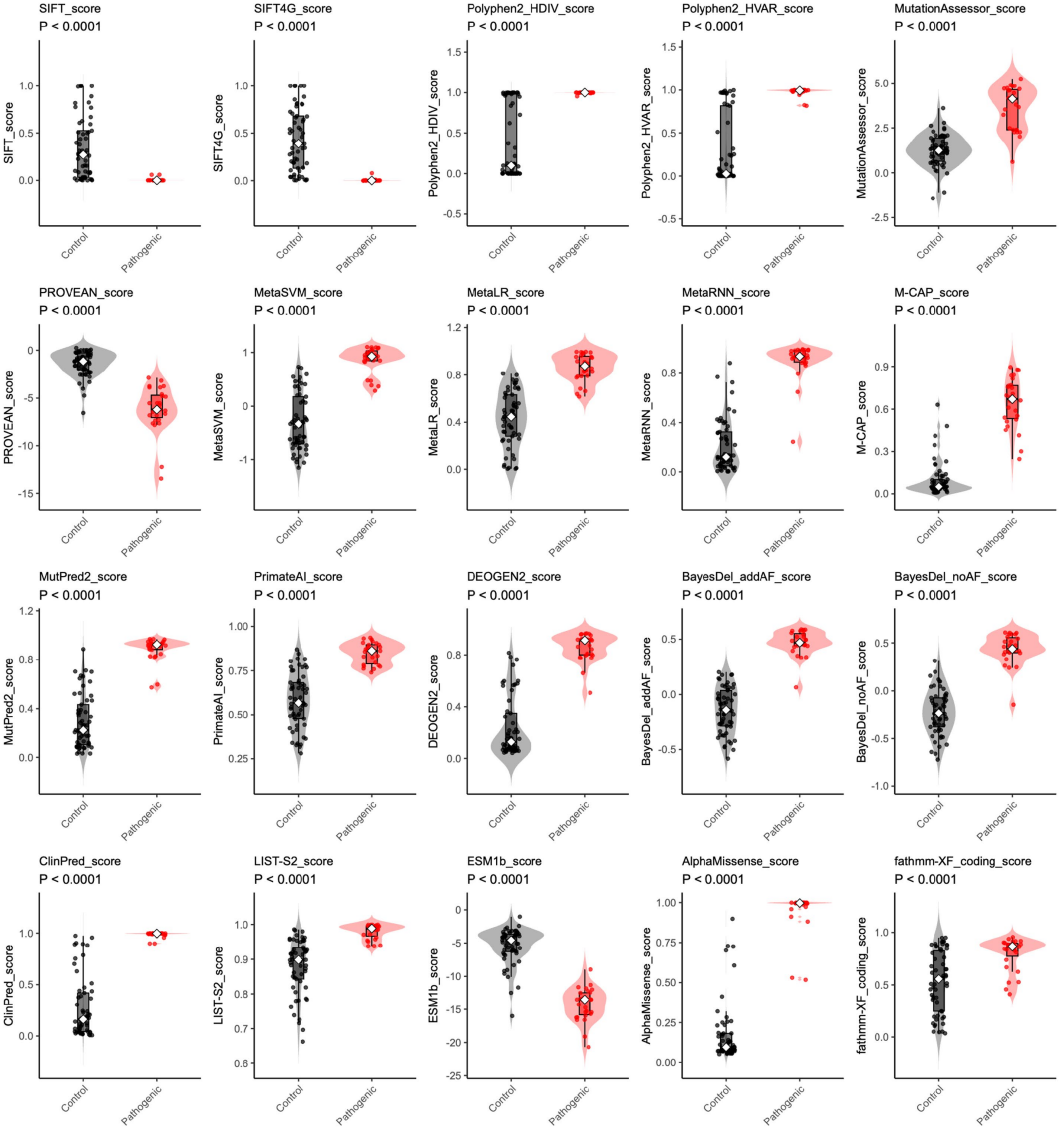
The distribution of *in silico* prediction raw scores for *CHD2* variants. For each algorithm (including SIFT, SIFT4G, Polyphen2_HDIV, Polyphen2_HVAR, MutationAssessor, PROVEAN, MetaSVM, MetaLR, MetaRNN, M-CAP, MutPred2, PrimateAI, DEOGEN2, BayesDel_addAF, BayesDel_noAF, ClinPred, LIST-S2, ESM1b, AlphaMissense, and fathmm-XF_coding), raw scores are shown for control (gray) and pathogenic (red) variants. Violin plots depict the score distribution. Higher rank scores indicate more deleterious predictions. Group differences were tested using a two-sided Wilcoxon rank-sum test; all comparisons were significant (*p* < 0.0001).

The prediction scores of 20 *in silico* tools on a continuous scale, applying a binary “cutoff point” and classifying variants as “pathogenic” or “control.” We, therefore, visualized the scores generated by these *in silico* tools in scatter plots. Most variant pathogenicity scores significantly differed between control and pathogenic groups (all *p*-value < 0.0001) (Figure 3). Interestingly, the SIFT, SIFT4G, PolyPhen2_HDIV, PolyPhen2_HVAR, ClinPred, and AlphaMissense scores displayed a distinct bimodal distribution, highlighting their ability to clearly distinguish pathogenic from control variants. In contrast, scores derived from other predictors exhibited broader and more continuous distributions, reflecting greater overlap between variant categories and suggesting varying levels of discriminative performance among prediction algorithms.

## 4 Discussion

In this study, we benchmarked 20 *in silico* predictors on 27 pathogenic and 57 control *CHD2* missense variants show substantial performance heterogeneity across tools. While MutPred2 achieved the highest balanced accuracy (≈95.4%). AlphaMissense, together with ClinPred and BayesDel_addAF, delivered near-perfect discrimination by ROC analysis. Given its consistently strong performance across multiple metrics, MutPred2 and AlphaMissense may be possible as the practical first line predictor for clinical triage of *CHD2* variants in DEE. This finding is particularly significant given the increasing number of *CHD2* variants identified in patients with DEE, as accurate prediction of variant pathogenicity is crucial for guiding clinical decision-making and treatment strategies.

The ranking of predictive tools differed significantly depending on the metric used to quantify performance and shown that no single predictive tool is best at all times. This is illustrated, for example, by the fact that MutPred2 had the best-balanced accuracy (0.954) and Matthews correlation coefficient (MCC = 0.918) while other tools, including ESM1b, PROVEAN, and ClinPred were superior in terms of sensitivity (1.000). Their perfect sensitivity was however limited by lower specificity and higher false positive rate. MutPred2 and AlphaMissense, on the other hand, performed well and generated a generally balanced result with regard to a number of key metrics, resulting in a top ranking in accuracy, correlation and discrimination. This elevates AlphaMissense to the status of a superb overall performer, which is to be trusted for overall balance between the identification of true pathogenic variants and minimum false positives.

We also assessed predictor performance from a ranking ability perspective and assessed ROC/AUC. This approach illuminates the principle that excellent discrimination is not tantamount to the best possible performance at a fixed or default threshold. A salient example in this case is M-CAP, which had perfect sensitivity, but poor specificity (0.167), and balanced accuracy (0.583) at its default cutoff, misclassifying 45 of 57 control variants. This shows that having the ability to rank pathogenic variants highly is not automatically correlated with good classification accuracy at a fixed, predetermined cutoff. Other predictors such as ClinPred (AUC = 0.995), AlphaMissense (AUC = 0.993), and BayesDel_addAF (AUC = 0.993), while also ranking well, also had excellent performance scores in terms of threshold classifications and therefore are the more useful predictors for direct clinical application.

An important consideration in the choice of the predictors is the trade-off between sensitivity and specificity, since our results show that it is often possible to obtain a high sensitivity at the expense of specificity. For example, ESM1b, PROVEAN and ClinPred identified all 27 pathogenic variants correctly (sensitivity = 1.000) but classified, respectively, 9, 10 and 11 of the benign/likely benign variants as pathogenic (specificity = 0.842, 0.825 and 0.807). This trade-off is particularly important for M-CAP and LIST-S2 in which case perfect sensitivity was also obtained, but specificities were very low (0.167 and 0.281, respectively). This very large false positive rate has great clinical significance as it could lead to unnecessary follow-up investigations or increased anxiety on the part of the patient. It would seem therefore that a balanced predictor or a combination of those that are complementary may be preferred for clinical use.

The performance patterns of these tools in our analysis offer important insights. The high accuracy of AlphaMissense and ESM-1b, for instance, strongly emphasizes that the structural integrity and sequence conservation of CHD2 proteins are critical determinants of their function, and that disruptions to these features are a major driver of pathogenicity. Their top-perform validated that methods focusing on these fundamental biochemical properties are highly effective. Additionally, ClinPred’s consistently high ranking in ROC evaluations underscores the importance and power of the “meta-predictor” strategy, which combines multiple, diverse algorithms to forge a more robust and accurate consensus. This suggests that future advancements may lie not only in developing novel individual predictors but also in intelligently integrating the strengths of existing ones.

This study indicates that the new predictors in use now, in particular those generated by deep learning and protein language models such as AlphaMissense (27) and ESM1b (50), tend to outperform many of the older generation predictors. This indicates the quick advancement in this area and goes some way to proving the need for frequent re-evaluation of those tools used in clinical practice. Guidelines such as those issued by the ACMG/AMP need to be continually evaluated in order to better reflect the capabilities of these predictors so that the best and most trustworthy evidence is always being used to classify variants. At the same time, it is well known that the ability of any particular predictor to accurately assess variants differs from gene to gene. This study gives a necessary gene specific assessment of *CHD2* and also draws attention to the vital importance of carrying out similar assessments on other clinically significant disease genes before any single predictor tool is used in a diagnostic context on a widespread basis.

The limitations of *in silico* tools must be appreciated and their predictions treated with caution. Predictive ability can be influenced by a number of factors, not least the quality and heterogeneity of the training datasets, methodology of prediction and the features of the variants studied. Validation of the predictions of these tools by experimental means, e.g., functional assays or clinical data is therefore essential in order to ascertain their reliability and accuracy. Furthermore, the classification is imbalanced between pathogenic and control variants. The imbalance reflected the real-world distribution of variants but may lead to inflated specificity value and potentially bias accuracy measurements. The study highlighted a focus on metrics insensitive to data imbalance, such as balanced accuracy, MCC rankings, and ROC/AUC results. However, sensitivity and specificity rankings serve only as suggestive indicators that necessitate prospective larger-scale clinical validation.

Missense variants are the most common variants in the real world, which may account for a major part of disease-causative variants. Recent studies showed missense variants is frequently associated with epilepsy in established genes of neurodevelopmental disorders, such as several genes reported in recent studies such as *ACTB* (51), *APC2* (52), *BCOR* (53), *CCDC22* (54), *DLG3* (55), *EP400* (56) *FRMPD4* (57), *GABRA1* (58), SZT2 (59), *TANC2* (60), and *KCNK4* (61). This study highlights the need for continued development of *in silico* pathogenicity predictors; as DEE genetics advances, more accurate and reliable tools are critical for clinical decision-making. Integrating these gene-specific functional datasets with curated clinical evidence provides the most direct way to assess and improve gene-specific predictor performance and reduce bias.

In conclusion, the study provides valuable insights into the performance of various *in silico* tools for predicting the pathogenicity of *CHD2* missense variants. By identifying MutPred2 and AlphaMissense as the first-line predictor, the study has demonstrated the potential of advanced bioinformatics methods to improve the accuracy and reliability of variant pathogenicity assessments. However, it is important to recognize the limitations of these tools and to validate their predictions using experimental methods. In the future, we look forward to the continued development and refinement of *in silico* tools to better serve the needs of the clinical community and improve patient care and outcomes.

## Data Availability

The original contributions presented in the study are included in the article/Supplementary material, further inquiries can be directed to the corresponding authors.

## Supplementary material

SUPPLEMENTARY FIGURE S1 All ROC curves. ROC curves were presented with AUC and 95% CI, including SIFT, SIFT4G, Polyphen2_HDIV, Polyphen2_HVAR, MutationAssessor, PROVEAN, MetaSVM, MetaLR, MetaRNN, M-CAP, MutPred2, PrimateAI, DEOGEN2, BayesDel_addAF, BayesDel_noAF, ClinPred, LIST-S2, ESM1b, AlphaMissense, and fathmm-XF_coding_rawscore.
