# Supplementary figures and images for: Epilepsy-associated CHD2 missense variants and optimization strategies for genetic diagnosis: a comparative analysis of algorithms
Source: Front Neurol. 2025 Nov 26;16:1729387. doi: 10.3389/fneur.2025.1729387 (PMC12689356; doi:10.3389/fneur.2025.1729387)

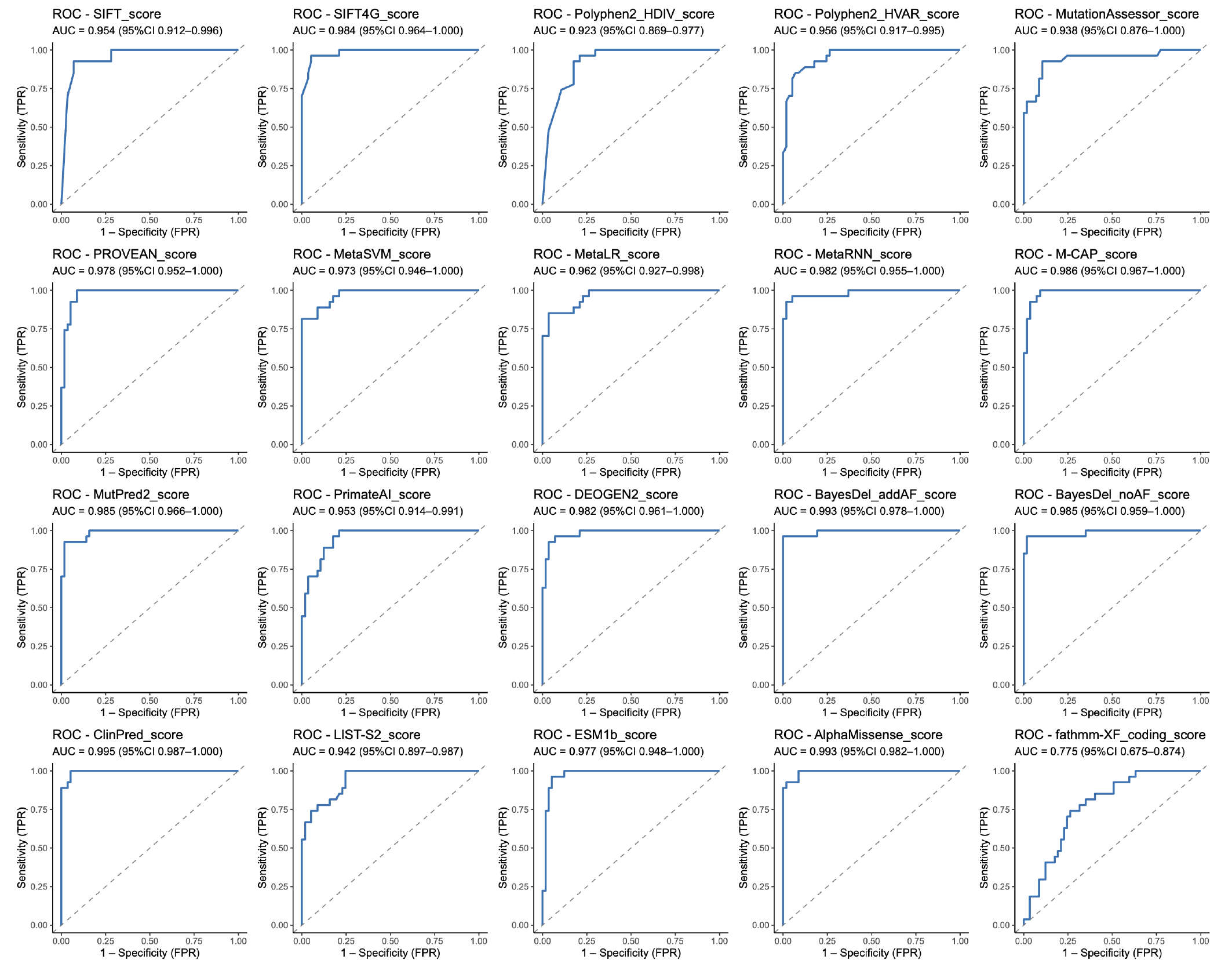

Supplement: SUPPLEMENTARY FIGURE S1 — All ROC curves. ROC curves were presented with AUC and 95% CI, including SIFT, SIFT4G, Polyphen2_HDIV, Polyphen2_HVAR, MutationAssessor, PROVEAN, MetaSVM, MetaLR, MetaRNN, M-CAP, MutPred2, PrimateAI, DEOGEN2, BayesDel_addAF, BayesDel_noAF, ClinPred, LIST-S2, ESM1b, AlphaMissense, and fathmm-XF_coding_rawscore. [file Image_1.TIF]
