# Supplementary material for: Epilepsy-associated CHD2 missense variants and optimization strategies for genetic diagnosis: a comparative analysis of algorithms
Source: Front Neurol. 2025 Nov 26;16:1729387. doi: 10.3389/fneur.2025.1729387 (PMC12689356; doi:10.3389/fneur.2025.1729387)
Supplement: Supplementary file 2 [file Table_1.DOCX]

**Table S1. List of likely pathogenic missense variants *CHD2* reported previous study.**

| Case | Variants (NM_001271) | Inheritance | Phenotype | MAF | ACMG scores | ACMG Classification | Ref. |
| --- | --- | --- | --- | --- | --- | --- | --- |
| 1 | c.1618G>T/p.Val540Phe | *De novo* | Short stature, ID, ADHD and autism | - | PS2+PM2 | LP | [1] |
| 2 | c.1642T>C/p.Trp548Arg | *De novo* | EE, moderate ID and DD | - | PS2+PM2 | LP | [2] |
| 3 | c.1854A>T/p.Glu618Asp | *De novo* | MD delay, speech delay, muscular, neurological and multisystem disease | - | PS2+PM2 | LP | [3] |
| 4 | c.1861C>T/p.Arg621Trp | *De novo* | Myoclonic atonic epilepsy | - | PS2+PM2 | LP | [4] |
| 5 | c.1934C>T/p.Thr645Met | *De novo* | Epilepsy, ASD, language deficit and repetitive behaviors | - | PS2+PM2 | LP | [5] |
| 6 | c.1942C>T/p.Pro648Ser | *De novo* | Seizures, NDD | - | PS2+PM2 | LP | [7] |
| 7 | c.2068C>T/p.His690Tyr | *De novo* | EECO, speech and language development delay, GDD and MD | - | PS2+PM2 | LP | [6] |
| 8 | c.2387T>C/p.Leu796Ser | *De novo* | Epilepsy, MD, LD and deficit, intellectual disability and hyperactivity | - | PS2+PM2 | LP | [7] |
| 9 | c.2408T>A/p.Leu803His | *De novo* | Epilepsy | - | PS2+PM2 | LP | [8] |
| 10 | c.2435T>C/p.Ile812Thr | *De novo* | ASD | - | PS2+PM2 | LP | [9] |
| 11 | c.2468T>C/p.Leu823Pro | *De novo* | EE, DD, severe ID and ASD | - | PS2+PM2 | LP | [2] |
| 12 | c.2537G>A/p.Arg846Gln | *De novo* | Epilepsy and DD | - | PS2+PM2 | LP | [10] |
| 13 | c.2609G>A/p.Gly870Asp | *De novo* | Epilepsy and DD | - | PS2+PM2 | LP | [10] |
| 14 | c.2636C>T/p.Ala879Val | *De novo* | Seizures, speech delay and ADHD | - | PS2+PM2 | LP | [11] |
| 15 | c.2644G>T/p.Val882Phe | *De novo* | Epilepsy and DD | - | PS2+PM2 | LP | [10] |
| 16 | c.2699G>A/p.Arg900Gln | *De novo* | Epilepsy & ID | - | PS2+PM2 | LP | [11] |
| 17 | c.2698C>G/p.Arg900Gly | *De novo* | Epilepsy, MD, LD and deficit, intellectual disability, autistic features, aggressive behavior and hyperactivity | - | PS2+PM2 | LP | [7] |
| 18 | c.2877C>A/p.Asn959Lys | *De novo* | EECO and GDD | - | PS2+PM2 | LP | [13] |
| 19 | c.3455G>A/p.Arg1152Gln | *De novo* | ADHD | - | PS2+PM2 | LP | [14] |
| 20 | c.3454C>G/p.Arg1152Gly | *De novo* | EECO | - | PS2+PM2 | LP | [15] |
| 21 | c.3454C>T/p.Arg1152Trp | *De novo* | Focal epilepsy, ID and ASD | - | PS2+PM2 | LP | [12] |
| 22 | c.3650T>A/p.Val1217Asp | *De novo* | EECO | - | PS2+PM2 | LP | [16] |
| 23 | c.3782G>T/p.Trp1261Leu | *De novo* | Epilepsy and NDD | - | PS2+PM2 | LP | [11] |
| 24 | c.3937C>G/p.Arg1313Gly | *De novo* | Epilepsy, photosensitive | - | PS2+PM2 | LP | [17] |
| 25 | c.3938G>C/p.Arg1313Pro | *De novo* | Developmental and epileptic encephalopathy | - | PS2+PM2 | LP | [18] |
| 26 | c.3947A>G/p.Tyr1316Cys | *De novo* | Neurological disorder and FS | - | PS2+PM2 | LP | [19] |
| 27 | c.4528G>A/p.Gly1510Arg | *De novo* | Epilepsy, MD, LD and aggressive behavior | - | PS2+PM2 | LP | [7] |

Abbreviations: ADHD, attention deficit hyperactivity disorder; ASD, autism spectrum disorder; D, *de novo*; DD, development delay; EE, epileptic encephalopathy; EECO, epileptic encephalopathy, childhood-onset; F, paternal; GDD, global developmental delay; ID, intellectual disability; JME, juvenile myoclonic epilepsy; LD, language delay; M, maternal; MD, motor delay; MAF, minor allele frequency from Genome Aggregation Database (controls); MI, maternal inheritance; NDD, neurodevelopmental disorder.

1. Cabrera-Salcedo, C., et al., *Targeted Searches of the Electronic Health Record and Genomics Identify an Etiology in Three Patients with Short Stature and High IGF-I Levels.* Horm Res Paediatr, 2019. **92**(3): p. 186-195.

2. Carvill, G.L., et al., *Targeted resequencing in epileptic encephalopathies identifies de novo mutations in CHD2 and SYNGAP1.* Nat Genet, 2013. **45**(7): p. 825-30.

3. van der Ven, A.T., et al., *Prevalence and clinical prediction of mitochondrial disorders in a large neuropediatric cohort.* Clin Genet, 2021. **100**(6): p. 766-770.

4. Routier, L., et al., *Exome sequencing findings in 27 patients with myoclonic-atonic epilepsy: Is there a major genetic factor?* Clin Genet, 2019. **96**(3): p. 254-260.

5. Lebrun, N., et al., *Autism spectrum disorder recurrence, resulting of germline mosaicism for a CHD2 gene missense variant.* Clin Genet, 2017. **92**(6): p. 669-670.

6. Trujillano, D., et al., *Clinical exome sequencing: results from 2819 samples reflecting 1000 families.* Eur J Hum Genet, 2017. **25**(2): p. 176-182.

7. De Maria, B., et al., *Expanding the genetic and phenotypic spectrum of CHD2-related disease: From early neurodevelopmental disorders to adult-onset epilepsy.* Am J Med Genet A, 2022. **188**(2): p. 522-533.

8. Piccolo, B., E. Gennaro, and F. Pisani, *A new CHD2 variant: not only severe epilepsy-a case report.* Acta Neurol Belg, 2022. **122**(6): p. 1653-1656.

9. Stranneheim, H., et al., *Integration of whole genome sequencing into a healthcare setting: high diagnostic rates across multiple clinical entities in 3219 rare disease patients.* Genome Med, 2021. **13**(1): p. 40.

10. Chen, J., et al., *CHD2-related epilepsy: novel mutations and new phenotypes.* Dev Med Child Neurol, 2020. **62**(5): p. 647-653.

11. Kaplanis, J., et al., *Evidence for 28 genetic disorders discovered by combining healthcare and research data.* Nature, 2020. **586**(7831): p. 757-762.

12. Niu, Y., et al., *Genetic and phenotypic spectrum of Chinese patients with epilepsy and photosensitivity.* Front Neurol, 2022. **13**: p. 907228.

13. Peng, J., et al., *Next-generation sequencing improves treatment efficacy and reduces hospitalization in children with drug-resistant epilepsy.* CNS Neurosci Ther, 2019. **25**(1): p. 14-20.

14. Alkelai, A., et al., *Genetic insights into childhood-onset schizophrenia: The yield of clinical exome sequencing.* Schizophr Res, 2023. **252**: p. 138-145.

15. Brunet, T., et al., *De novo variants in neurodevelopmental disorders-experiences from a tertiary care center.* Clin Genet, 2021. **100**(1): p. 14-28.

16. French, C.E., et al., *Refinements and considerations for trio whole-genome sequence analysis when investigating Mendelian diseases presenting in early childhood.* HGG Adv, 2022. **3**(3): p. 100113.

17. Galizia, E.C., et al., *CHD2 variants are a risk factor for photosensitivity in epilepsy.* Brain, 2015. **138**(Pt 5): p. 1198-207.

18. Shin, S., et al., *Genetic Diagnosis of Children With Neurodevelopmental Disorders Using Whole Genome Sequencing.* Pediatr Neurol, 2023. **149**: p. 44-52.

19. Jiao, Q., et al., *The combination of whole-exome sequencing and copy number variation sequencing enables the diagnosis of rare neurological disorders.* Clin Genet, 2019. **96**(2): p. 140-150.
